# Age-related factors influencing the occurrence of undernutrition in northeastern Ethiopia

**DOI:** 10.1186/s12889-015-1490-2

**Published:** 2015-02-07

**Authors:** Abraham Degarege, Elifaged Hailemeskel, Berhanu Erko

**Affiliations:** Aklilu Lemma Institute of Pathobiology, Addis Ababa University, P. O. Box 1176, Addis Ababa, Ethiopia; Department of Biology, College of Natural Sciences, Wollo University, P. O. Box 1145, Dessie, Ethiopia

**Keywords:** Undernutrition, Age pattern, Socioeconomy, Blood type, Ethiopia

## Abstract

**Background:**

Undernutrition is a major public health problem on the globe particularly in the developing regions. The objective of the current study was to assess the prevalence of undernutrition in different age groups and examine the relationship of the disease to parasitic and socioeconomic factors among communities in Harbu Town, northeastern Ethiopia.

**Methods:**

Stool samples of the study participants were examined for intestinal helminth infections using the Kato-Katz method. Blood specimens were diagnosed for *Plasmodium* infection using CareStartTM Malaria Pf/Pv Combo test. The blood type was determined from blood samples using antisera A and antisera B. In addition, the height and weight of the study participants was measured and information about their socioeconomic and sociodemographic characteristics was collected.

**Results:**

Out of 484 individuals examined, 31.8% were undernourished and 32.0% were infected with intestinal helminths. The odds of undernutrition significantly decreased with an increase in the age of individuals. The prevalence of undernutrition in adults was significantly higher in males than in females and in those who had latrines than in those who did not have the facility. The odds of undernutrition in the 5 to 19 years age group was significantly higher in those who did not wash their hands before eating than in those who did. The prevalence of undernutrition in children younger than five years was significantly lower in those whose families were educated and had less than 5 family size compared to those with illiterate families and family size of greater than 5, respectively. However, the prevalence of undernutrition was similar in individuals who were infected and not infected with intestinal helminths. The intensity of *Schistosoma mansoni* infection was significantly higher among individuals of blood type A compared to those of type O.

**Conclusions:**

Prevalence of undernutrition was higher in children than in adults and the association of sex and socioeconomic factors with undernutrition showed variation with age. However, helminth infection was not related with undernutrition in all age groups.

## Background

Undernutrition is a major public health problem on the globe particularly in the developing regions [1]. About 870 million people in the world and slighter fewer than 15% of the population in the developing regions were estimated to be undernourished in 2010-12 [1]. Undernutrition was responsible for the death of 1.5 million children and women in the world in 2010 [2]. The disease is prevalent in Ethiopia [3-6].

Different biological, socioeconomic and environmental factors are reported to affect the dietary intake and nutrient absorption capacity of individuals [7-9]. Helminths are also implicated in affecting host nutrition [10-12]. For example, *S. manosni* and hookworm infections could cause high blood and micronutrients loss from the intestinal wall [10,11]. *Ascaris lumbricoides* (*A. lumbricoides*) and *Trichuris trichiura* (*T. trichiura*) may also cause physiological abnormality of the intestinal wall leading to reduced food intake and nutrient absorption [10,11].

As the prevalence and intensity of intestinal helminth infection as well as the biology, socioeconomic and exposure status to environmental risk factors in humans could vary with age, the relationship of undernutrition with these factors could show an age-related patterns [13,14]. However, the strength and age patterns of association of undernutrition with biological, social, economic, environmental and parasitic factors remain uncertain [7-9,12,13]. In addition, most previous studies in Ethiopia assessed the effect of helminth infection on undernutrition in school age children and febrile adult patients [3-5]. However, the information on apparently healthy looking individuals of different age groups would be more helpful to design appropriate intervention strategies to control the disease. Although major risk factors for undernutrition have been investigated in different regions [7-9,12,13], individual factors potentially change in specific areas, age group and over time. Thus, determining risk factors of undernutrition in specific regions and age groups will be vital to design appropriate intervention measures against the problem.

Moreover, few studies have suggested evolutionary relationship between *Schistosoma mansoni* (*S. mansoni*) infection and blood type [15,16]. Supporting this notion, an increased prevalence of *S. mansoni* infection and related morbidity was reported in people with blood types A [17,18] and B [19]. However, others documented insignificant impact of blood type on *S. mansoni* infection [20,21]. The objective of the current study was to assess the age patterns of relationship of undernutrition with biological, socioeconomic, household and parasitic factors among communities in Harbu Town, northeastern Ethiopia.

## Methods

### Study area and population

A community-based cross-sectional study was conducted in Harbu Town, northeastern Ethiopia, in May and June 2013. The town is located 355 Km northeast of Addis Ababa, in the Amhara Regional State. It lies at an elevation of 1842 meters above sea level, with a longitude of 3943′59.880"E and latitude of 114′59.880"N. The town is divided into 8 subunits and has a population of 20,000 and 6367 households. Intestinal helminth infections are known to be prevalent in the area [22].

### Sample size and sampling procedure

Since similar studies were not previously conducted in the current study area, we considered a 50% prevalence of undernutrition among communities to estimate the sample size. Assuming random sampling and an approximate normality in the distribution of the proportion of events, and taking the sampling error as 5%, or a 95% confidence interval (CI), the lowest number of individuals that would be representative of the communities in Harbu Town was 384. Considering a designing effect of 1.3, the sample size for the nutritional survey was estimated to be 499.

A total of 484 individuals were selected for the study. Since the average number of people living in a house in the town was 3 (population size/number of households in the town), a total of 161 households (484/3) were visited. The number of households visited was proportionally divided among the 8 subunits based on the size of households in each subunit. Then, specific households where samples were collected were decided after determining the interval between any two households. The size of the interval was decided by dividing the total number of household in each of the subunit by the number of households allocated for each subunit. All voluntary family members in the selected houses, who were permanent residents in the town, provided stool samples and their height and weight were measured. All adolescents and adults living in the households were interviewed for information about income level, education, employment, marital status, house and latrine floor, family size, drinking water source, hand washing habit, ethnicity and religion. Parents/guardians were also asked to provide information about the child on habits such as hand washing.

### Stool diagnosis for intestinal helminths

Two Kato-Katz slides were prepared from each stool sample and examined for intestinal helminths using microscope in the field within 45 minutes of the smear preparation [23]. Hookworm eggs were counted in the field but counting for other intestinal helminth species was done in the laboratory at Aklilu Lemma Institute of Pathobiology, Addis Ababa University. The average values of the egg per gram of the two slide reading were considered to estimate the intensity of infection for each parasite species.

### Blood diagnosis for *Plasmodium*

About 5 μl finger prick blood was taken from every participant who gave stool sample and checked for *Plasmodium* infection using CareStartTM Malaria Pf/Pv Combo test following manufacturer’s instruction (Access Bio, Inc. NJ USA).

### Blood group determination

About 2 drops of finger prick blood was taken from every participant who gave stool sample and placed at two different places on a slide and ABO blood type was determined using antisera A and antisera B (Tulip Diagnostics (p) Ltd, Verna, India).

### Nutritional status assessment

Height and weight of the study participants were measured in barefoot and light clothing using digital balance and wooden board fixed with plastic tape, respectively. Then, z-scores were determined for children using anthro (age ≤ 5 years) and anthro-plus (age 5.1 to 19 years) softwares [24,25] and body mass index (Kg/m^2^) was calculated for adults. Children were grouped as undernourished when either underweight (Z of weight-for-age or body mass index for age < −2), stunted (Z of height-for-age < −2) or wasted (Z of weight-for-height < −2) [26]. Adults were classified as undernourished when their BMI was less than 18.5 [27].

### Data analysis

Data were double entered in excel sheet and analyzed in STATA (version 11). Bivariate and multivariate logistic regression analysis were used to quantify the impact of intestinal helminth infection, socio-demographic, socioeconomic, household, hand washing habit, religious and ethnic factors on the prevalence of undernutrition. Multivariate logistic regression analysis was also used to test the impact of blood type on prevalence of *S. mansoni* infection and related undernutrition. Multivariate linear regression analysis was used to quantify the impact of blood type on the intensity of *S. manosni* infection. 95% confidence interval was calculated for the regression coefficients and odds ratio values. Values were considered significant at *P* < 0.05.

### Ethical clearance

The protocol for the present study obtained ethical approval from the Ethical Clearance Committee of the Department of Biology, Wollo University, Ethiopia. Permission to conduct the study was also obtained from the administrative of the town and the head of the Harbu Health Center. Only voluntary individuals after giving written consent participated in the study. Parents or guardians were also asked written consent for their willingness about the participation of their children in the study. Individuals who were positive for intestinal helminths were treated with appropriate anti-helminthic drugs.

## Results

A total 484 individuals (mean age = 22.47; age range: 1 to 80 years; male = 36.6%) living in Harbu Town were involved in the study (Table 1). More than 90% of the community in the current study area were self-employed, live in house with mud floor, Muslims religious followers, Amhara in ethnicity, had toilet and used piped water for drinking. Majority (>75%) of the study participants wash their hands before eating and after using latrine.

**Table 1:** **Sociodemographic and socioeconomic characteristics of the study participants in Harbu Town, northeastern Ethiopia, May and June 2013**

**Characteristics**	**Categories**	**Frequency**	**Percent**
Marital status	Married	281	78.7
	Divorced	27	5.6
	Widowed	55	11.4
	Single	121	4.3
Family size	≤5	318	65.7
	>5	166	34.3
Ethnicity	Amhara	471	97.3
	Tigre	7	1.2
	Other	7	1.4
Religion	Orthodox	34	7.0
	Muslim	443	91.5
	Protestant	7	1.5
Educational status of mother	Cannot read and write	274	56.6
	No formal education	13	2.7
	Grade 1–6 completed	113	23.4
	Grade 7–12 completed	74	15.3
	College and above	10	2.0
Monthly income (in Ethiopian Birr)	≤250	101	21.3
	251-500	266	54.9
	501-1000	88	18.2
	≥1001	27	5.6
Presence of toilet at home	No	8	1.7
	Yes	476	98.4
Type of floor house	Mud	437	90.3
	Cemented	40	8.3
	Other	7	1.5
Hand washing habit after latrine	Yes	375	77.5
	No	109	22.5
Hand washing habit before eating	Yes	379	78.3
	No	105	21.7
Source of drinking water	Piped*	429	88.6
	Well	1	0.2
	River, pond or lake	52	10.7
	Bottled water	2	0.4

Piped water*: pressurized and treated ground water reaching to people for use through pipes or tubes.

### Prevalence of undernutrition

About 31.8% of the participants were undernourished and 32.0% were infected with intestinal helminth (Table 2). However, none of the study participants was found positive for *Plasmodium* infection. Prevalence of underweight among the study participants was 23.8% (115). About 25.1% (64) of the individuals in the 1 to 19 years age group were stunted and 16% (8) of the children younger than five years of age were wasted. Prevalence of undernutrition was greater in individuals of younger than 19 years (42.35%) compared to those older than 19 years (20.52%) (p < 0.01). Prevalence of stunting in school age children was higher in males than in females (x^2^ = 4.61, p = 0.032).

**Table 2:** **Prevalence of undernutrition among communities in Harbu Town, northeastern Ethiopia, May and June 2013**

**Age in years**	**Sex**	**Number examined**	**Percent wasted (mean WHZ)**	**Percent stunted (mean HAZ)**	**Percent underweight (mean BAZ or mean BMI)**	**Percent undernourished**
<5	Females	21	14.3 (−0.4)	23.8 (−0.9)	23.8 (−0.3)	38.10
	Males	29	17.2 (0.7)	41.4 (−1.7)	27.6 (1.0)	55.17
	Total	50	16.0 (0.2)	34.0 (−1.4)	26.0 (0.4)	48.00
5-19	Females	128	NA	18.0 (−1.1)	24.2 (−0.7)	36.72
	Males	77	NA	30.2 (−1.2)	30.2 (−0.9)	48.05
	Total	205	NA	23.0 (−1.2)	26.8 (−0.7)	41.98
>19	Females	158	NA	NA	18.4 (21.3)	18.35
	Males	71	NA	NA	25.4 (20.7)	25.35
	Total	229	NA	NA	20.5 (21.1)	20.52

WHZ = Z-scores for Weight-for-length/height-calculated when age <5 years.

WAZ = Z-scores for Weight-for-age- calculated when age from 1 to 10 years.

HAZ = Z-scores for Length/height-for-age- calculated when age from 1 to 19 years.

BAZ = Z-scores for BMI-for-age- calculated when age from 1 to 19 years.

BMI = Body Mass Index- calculated when age greater than >19 years.

NA = not applicable.

### Relationship of nutritional status with biological, parasitic, socioeconomic and household factors

Information about the relationship of nutritional status with biological, parasitic, socioeconomic and environmental factors without stratifying the data into different age groups (i.e. cumulative analysis) is summarized in Table 3. The odds of undernutrition was higher in males than in females (adjusted Odds Ratio, AOR = 2.13, 95% CI = 1.36, 3.34) but significantly decreased with an increase in the age of individuals (AOR = 0.98, 95% CI = 0.97, 0.99). The chance of undernutrition was about 3 (AOR = 2.65, 95% CI = 1.31, 5.37) and 2 (AOR = 2.15, 95% CI = 1.35, 3.41) times higher among children of age younger than 5 years and in the 5 to 15 years, respectively compared to individuals of older than 15 years. However, the difference in prevalence of undernutrition among the study participants was not significant when compared between individuals who were infected and none-infected with intestinal helminths. Similarly, the prevalence of undernutrition was comparable among individuals with different education, income, employment, marital, house and latrine floor, drinking water source and hand washing status.

**Table 3:** **Relationship of nutritional status with biological, parasitic, socioeconomic and environmental factors in communities living in Harbu Town, northeastern Ethiopia, May and June 2013**

**Variable**	**Categories**	**Number**	**Percent undernourished**	Adjusted odds ratio of undernutrition*
Intestinal helminth	Not infected	329	29.27	1
Infection status	*S. mansoni*	129	37.98	2.21 (0.43, 11.33)
	*A. lumbricoides*	7	28.57	0.92 (0.11, 7.71)
	*H. nana*	20	40.00	2.37 (0..46, 12.26)
	Hookworm	5	60.00	4.08 (0.48, 34.59)
	*T. trichiura*	2	Not applicable	Not applicable
	*E. vermicularis*	7	42.86	1.82 (0.30, 10.83)
	Any helminth	155	36.77	0.49 (0.09, 2.66)
Age in years	<5	50	48.00	1
	5-15	189	39.36	00.86 (0.43, 1.73)
	>15	245	22.45	0.38 (0.19, 0.79)
Age as a continuous	Age as a continuous	384	31.68	0.98 (0.97, 0.99)
Sex	Female	307	27.04	1
	Male	177	39.77	20.13 (1.36, 3.34)

*Adjusted for monthly income, education level, type of occupation, family size, marital status, religious group, ethnicity, type of house floor, type of latrine floor, hand washing habit after latrine, hand washing habit before eating and cooking, source of drinking water, place of bath. **Note:** the difference in prevalence of undernutrition was not significant when compared among groups under these variables and the data for these are not shown in Table 3.

### Pre-school age children

The prevalence of undernutrition in children younger than five years was significantly lower in those whose families were educated at primary, secondary or tertiary level than those whose families were illiterate or did not have formal education (AOR = 0.02, 95% CI = 0.00, 0.69). Prevalence of undernutrition in children younger than five years was lower in those with a family less than 5 people compared to that with a family greater than 5 (AOR = 0.02, 95% CI = 0.00, 0.93). However, the difference in prevalence of undernutrition in pre-school age children was not significant when compared between those who were infected and not-infected with helminths, males and females or families of different income, martial, religious, ethnic, occupation and household status in the multivariate regression analysis (data not shown).

### School age children and adolescents

The odds of undernutrition was greater for those who did not wash their hands before eating than those who did (AOR = 30.27, 95% CI = 1.02, 898.03) among individuals in the 5 to 19 years age group. However, the difference in prevalence of undernutrition in individuals of age 5 to 19 years was not significant when compared between those who were infected and not infected with helminths, males and females or families of different income, education, family size, martial, religious, ethnic, occupation and household status in the multivariate regression analysis (data not shown).

### Adults

The prevalence of undernutrition in adults was greater in males than in females (AOR = 3.57, 95% CI = 1.35, 9.43) and in those who had latrine than in those who did not have latrine (AOR = 15.69, 95% CI = 1.15, 213.40) in the multivariate regression model. However, the difference in prevalence of undernutrition was comparable between those who were infected and not infected with helminths or those with different occupation, education, income, martial, drinking water source, hand washing, religious and ethnic status in the multivariate regression analysis (data not shown).

### *S. mansoni* infection and blood type

Information about the association of blood type with prevalence and intensity of *S. mansoni* infection and related undernutrition is summarized in Table 4. The intensity of *S. mansoni* infection was significantly higher among individuals of blood type A compared to those of blood type O (β = 2404.6, 95% CI = 86.9, 4722.3). However, the odds of *S. mansoni* infection were comparable among individuals of different blood groups. Similarly, the prevalence of undernutrition was not different when compared among *S. mansoni* infected individuals of different blood types.

**Table 4:** **Association of blood type with prevalence and intensity of** ***S. mansoni*** **infection and related undernutrition among communities in Harbu Town, northeastern Ethiopia, May and June 2013**

**Blood group**	**Number examined**	**Percent infected**	**Mean egg per gram**	AOR of infection*	Adjusted mean epg difference (β)*	AOR of undernutrition among infected cases*
O	165	23.03	2379.7	1	1	1
A	167	28.74	3912.0	1.14 [0.65, 1.98]	2404.6 [86.9, 4722.3]	0.40 [0.12, 1.29]
B	113	29.20	3403.6	1.10 [0.61, 1.99]	1206.4 [−1301.9, 3714.8]	0.67 [0.19, 2.28]
AB	38	23.68	1664.0	0.74 [0.29, 1.92]	265.6 [−3923.9, 4455.2]	0.66 [0.10, 4.24]

*Adjusted for age, sex, amount of monthly income, education level, type of occupation, family size, marital status, religious group, ethnicity, type of house floor, type of latrine floor, hand washing habit after latrine, hand washing habit before eating and cooking, source of drinking water, place of bath.

AOR = adjusted odds ratio epg = egg per gram.

## Discussion

Out of 484 individuals examined, 31.8% were undernourished and 32.0% were infected with intestinal helminths. The prevalence of undernutrition in the current study decreased with an increase in the age of individuals and was lower in females than males. Several studies in low-income sub-Saharan African countries also documented a higher prevalence of undernutrition in males and children compared to females and adults, respectively [5,13,28-30]. The young children may have bacterial infections resulting in diarrhea which could in turn cause high nutrient loss from their body. On the other hand, older age individuals will usually consume varieties of foodstuffs thereby preventing undernutrition. The country or community specific characters related with culture in the area could also be responsible for the increased prevalence of undernutrition in males in the current study. For example, in the current study area males are more responsible members of the family in generating income and keeping the welfare of the family. As a result, males in the area are more mobile and engaged in activities which could make them lose grater amount of energy. Indeed, studies reported a regional and country specific variation about the difference in prevalence of undernutrition between males and females [31]. The studies documented a higher prevalence of undernutrition in males than in females in sub-Saharan Africa and the reverse in the case of South/Southeast Asia and Latin America [31].

In the current study, the relationship between sex and undernutrition was observed in adults only. Male and female children and adolescents in the study area usually have similar activities (i.e. playing habit) and most are students, and hence a similar level of undernutrition between the two sexes However, a significant variation in terms of activities and responsibilities exist between males and females in the case of adults which could have resulted in difference in prevalence of undernutrition observed. In India, sex specific variation in prevalence of undernutrition was found at all stages of life [32]. Perhaps, there could have been other cultural reasons (e.g. gender discrimination) that may favor one sex but prevent the other from access to sufficient food and health care at all stages of life.

A decrease in the prevalence of undernutrition among preschool age children, who had educated parents and a family with less than 5 members, was observed in the current study. Other previous studies also documented a lower prevalence of undernutrition in children whose parents had better education status and lower number of family people [33,34]. The level of parental practices towards provision of balanced diet and heath/sanitation cares for their children is expected to increase as the level of education status of the parents increase and the number of family members decrease [33,34]. However, the reason why this was not maintained in school age children and in adults could be due to the fact that older age children and adults could give care for themselves. Adults and older age children may eat and keep their sanitation or visit heath centers more often even when they are uneducated or have big family size.

Washing hands before eating was related to a decrease in prevalence of undernutrition in school age children. However, this relationship did not exist in pre-school age children and adults. Unlike preschool age children and adults, school age children living in the area might play in dusts and mud. As a result, washing of hands in this age group will be more advantageous as it will decrease the chance of bacterial and parasitic infection. This in turn decreases nutrients loss due to infection.

The prevalence of undernutrition was similar between individuals who were infected and not infected with helminths. A study documented also lack of correlation of intestinal helminth infection with undernutrition [35]. The effect of helminths on the nutritional status of a host is more pronounced when infections are chronic and heavy [10,36]. All cases of *A. lumbricoides* and *T. trichiura* infection in the present study were of light intensity. However, all *S. manosoni* infections showed heavy intensity. Perhaps, *S. mansoni* infection in the study participants might be acute. Individuals who were not infected with intestinal helminths might have been infected with other bacterial, viral or chronic infections that could have negatively affected their nutritional status.

The prevalence of undernutrition in the current study was also similar when compared among individuals of different income level, employment status, house floor, ethnic and religious groups as previously reported in some studies [7,8]. Majority (>90%) of the community in the current study area were self employed, live in house with mud floor, were Muslims and Amhara in ethnicity. This might have hindered from detecting significant statistical differences. Furthermore, the life style of the community (e.g. culture and ethinic customs), availability and cost of foods in the area could also explain the current finding.

The intensity of *S. mansoni* infection was significantly higher among individuals of blood type A compared to those of blood type O. A study in Zimbabwe also documented a higher intensity of *S. mansoni* infection in children of blood type A compared to those with O type [18]. Indeed, studies showed structural similarity between substances (e.g. N-acetyl-D-galactose polysaccharides) in *S. mansoni* and blood type A [15,16]. This might help *S. mansoni* to mask its antigens and escape the host immune in blood type A individuals thereby increasing survival of the parasite in the host. However, studies involving larger samples in high prevalence and intensity areas for *S. mansoni* infection will be important to make firm conclusion about the impact of ABO blood type on prevalence of *S. mansoni* infection and other related morbidities. Furthermore, there is a need to make detail investigation as to how blood type A will increase susceptibility of the host to increased intensity of *S. mansoni* infection.

## Conclusions

In conclusion, prevalence of undernutrition was higher in children than in adults and the association of sex and socioeconomic factors with undernutrition varies with age. However, helminth infection was not associated with undernutrition in all age groups. Blood type A was associated with higher intensity of *S. mansoni* infection. Further study on how blood type A could lead to increased intensity of *S. mansoni* infection in infected individuals is necessary.
